# Supplementary material for: Comparative Proteomics of Peritrophic Matrix Provides an Insight into its Role in Cry1Ac Resistance of Cotton Bollworm Helicoverpa armigera
Source: Toxins (Basel). 2019 Feb 2;11(2):92. doi: 10.3390/toxins11020092 (PMC6409725; doi:10.3390/toxins11020092)
Supplement: Supplementary file 1 [file toxins-11-00092-s001.pdf]

Supplementary Materials: Comparative Proteomics of Peritrophic Matrix Provides an Insight into its Role in Cry1Ac Resistance of Cotton Bollworm *Helicoverpa armigera*

Minghui Jin, Chongyu Liao, Swapan Chakrabarty, Kongming Wu and Yutao Xiao

Table S1. KEGG pathways of P-value < 0.05 in XJ10 compared with XJ.

KEGG pathway	Pathway ID	Gene number	*P*-value
Ribosome biogenesis in eukaryotes	ko03008	28	2.47E-11
Metabolism of xenobiotics by cytochrome P450	ko00980	27	2.90E-07
Drug metabolism - cytochrome P450	ko00982	25	3.31E-07
Porphyrin and chlorophyll metabolism	ko00860	19	1.52E-05
Retinol metabolism	ko00830	15	0.000260309
Ascorbate and aldarate metabolism	ko00053	15	0.00041008
Pentose and glucuronate interconversions	ko00040	19	0.00054811
Drug metabolism - other enzymes	ko00983	18	0.001095362
Starch and sucrose metabolism	ko00500	16	0.004122616
Arachidonic acid metabolism	ko00590	8	0.01211841
Nicotinate and nicotinamide metabolism	ko00760	6	0.01614292
Protein processing in endoplasmic reticulum	ko04141	22	0.02472588
Vitamin B6 metabolism	ko00750	2	0.03268697
Glutathione metabolism	ko00480	13	0.03509358
One carbon pool by folate	ko00670	4	0.04403044

Table S2. Proteins were identified and quantified in Peritrophic matrix.

Description	Symbol	Peptides number
PREDICTED: UPF0586 protein C9orf41 homolog [Papilio polytes]	XM_021325089.1	1
PREDICTED: hepatoma-derived growth factor-related protein 2 [Papilio machaon]	XM_021325095.1	1
PREDICTED: histone H3.3 isoform X1 [Nothobranchius furzeri]	XM_021338021.1	1
Gag-pol polyprotein [Operophtera brumata]	XM_021325299.1	1
elongation factor 1-gamma [Papilio xuthus]	XM_021325364.1	1
PREDICTED: leukocyte surface antigen CD53-like isoform X1 [Papilio machaon]	XM_021325436.1	2
PREDICTED: neural cell adhesion molecule 2-like isoform X1 [Bombyx mori]	XM_021325537.1	1
cGMP-dependent protein kinase, isozyme 1 [Papilio xuthus]	XM_021325660.1	1
insect intestinal mucin 2, partial [Helicoverpa armigera]	XM_021326099.1	10
ionotropic receptor [Ostrinia furnacalis]	XM_021326308.1	2
PREDICTED: transcription factor TFIIIB component B'' homolog [Papilio xuthus]	XM_021326460.1	1
lipase [Helicoverpa armigera]	XM_021326676.1	4
PREDICTED: sodium-coupled monocarboxylate transporter 1 [Bombyx mori]	XM_021327183.1	1
serine protease inhibitor 5 [Helicoverpa armigera]	XM_021327264.1	1
Karyopherin beta 3 [Pararge aegeria]	XM_021327300.1	1
prophenol oxidase activating enzyme 3 [Spodoptera litura]	XM_021327314.1	1
PREDICTED: guanine nucleotide-binding protein G(o) subunit alpha isoform X2 [Papilio xuthus]	XM_021327420.1	1
wunen, partial [Pararge aegeria]	XM_021327456.1	1
CLUMA_CG005324, isoform A [Clunio marinus]	XM_021327707.1	4
rRNA 2'-O-methyltransferase fibrillarin [Papilio machaon]	XM_021327734.1	1
PREDICTED: coiled-coil domain-containing protein 97 [Amyelois transitella]	XM_021327892.1	1
PREDICTED: synaptic vesicle glycoprotein 2A-like [Bombyx mori]	XM_021327898.1	1
PREDICTED: E3 ubiquitin-protein ligase HECTD1 [Amyelois transitella]	XM_021328040.1	2
60S acidic ribosomal protein P2 [Spodoptera frugiperda]	XM_021328059.1	1
PREDICTED: LOW QUALITY PROTEIN: carboxypeptidase B-like [Bombyx mori]	XM_021328067.1	4
PREDICTED: vacuolar ATPase assembly integral membrane protein VMA21 homolog [Amyelois transitella]	XM_021328173.1	1
diacylglycerol O-acyl transferase [Manduca sexta]	XM_021328350.1	1
PREDICTED: unconventional myosin-XV isoform X2 [Bombyx mori]	XM_021328621.1	1
PREDICTED: cytochrome b5-like isoform X2 [Papilio polytes]	XM_021328653.1	1
Symplekin [Papilio xuthus]	XM_021328674.1	1
methionine-rich storage protein [Spodoptera litura]	XM_021328869.1	8
PREDICTED: probable transaldolase [Amyelois transitella]	XM_021328990.1	1
PREDICTED: monosaccharide-sensing protein 1-like isoform X1 [Papilio xuthus]	XM_021329072.1	1
PREDICTED: dynein heavy chain 8, axonemal [Amyelois transitella]	XM_021329100.1	1
ribosomal protein S27A [Spodoptera frugiperda]	XM_021329136.1	2
sarco/endoplasmic reticulum calcium ATPase [Bombyx mori]	XM_021329296.1	1
PREDICTED: E3 ubiquitin-protein ligase Bre1 [Bombyx mori]	XM_021329346.1	1
PREDICTED: tubulin alpha-1 chain-like [Amyelois transitella]	XM_021338879.1	1
63 kDa sperm flagellar membrane protein [Papilio xuthus]	XM_021329558.1	1
diazepam-binding inhibitor [Helicoverpa armigera]	XM_021329625.1	1
lysozyme precursor [Helicoverpa zea]	XM_021330615.1	1
carboxypeptidase precursor [Helicoverpa armigera]	XM_021330765.1	1
carboxypeptidase B precursor [Helicoverpa zea]	XM_021330831.1	2
carboxypeptidase A [Helicoverpa armigera]	XM_021330834.1	2
carboxypeptidase [Helicoverpa armigera]	XM_021330844.1	1
carboxypeptidase [Helicoverpa armigera]	XM_021330848.1	3
serine protease 24 [Mamestra configurata]	XM_021330937.1	2
Mini-chromosome maintenance complex-binding protein [Papilio xuthus]	XM_021331050.1	1
isochorismatase domain containing protein [Bombyx mori]	XM_021331129.1	1
ribosomal protein L23 [Bombyx mori]	XM_021331155.1	2
neutral lipase [Helicoverpa armigera]	XM_021331172.1	2
neutral lipase [Helicoverpa armigera]	XM_021331174.1	2
neutral lipase [Helicoverpa armigera]	XM_021331175.1	2
PREDICTED: proton-coupled amino acid transporter 4 isoform X1 [Plutella xylostella]	XM_021331270.1	1
C5197 [Bicyclus anynana]	XM_021331278.1	1
PREDICTED: twitchin [Amyelois transitella]	XM_021331369.1	2
PREDICTED: Krueppel homolog 2 [Amyelois transitella]	XM_021331414.1	1
Extracellular domains-containing protein CG31004 [Papilio machaon]	XM_021331713.1	1
PREDICTED: calnexin [Bombyx mori]	XM_021332065.1	1
PREDICTED: CTL-like protein 2 isoform X3 [Papilio machaon]	XM_021332162.1	1
PREDICTED: macrophage migration inhibitory factor-like [Amyelois transitella]	XM_021335062.1	1
PREDICTED: sodium/potassium/calcium exchanger 3-like [Papilio xuthus]	XM_021332356.1	1
PREDICTED: surfeit locus protein 4 homolog [Papilio polytes]	XM_021332409.1	1
heat shock protein [Helicoverpa armigera]	XM_021332476.1	5
alpha-amylase [Helicoverpa armigera]	XM_021332568.1	1
-	XM_021332684.1	1
chitin-binding protein, partial [Helicoverpa armigera]	XM_021332791.1	1
calreticulin precursor [Papilio xuthus]	XM_021332929.1	2
PREDICTED: V-type proton ATPase 21 kDa proteolipid subunit-like [Plutella xylostella]	XM_021332932.1	1
PREDICTED: protocadherin-like wing polarity protein stan [Amyelois transitella]	XM_021332965.1	3
PREDICTED: 27 kDa hemolymph protein-like [Papilio machaon]	XM_021332985.1	2
PREDICTED: dentin sialophosphoprotein isoform X1 [Papilio xuthus]	XM_021333267.1	1
PREDICTED: peroxisome biogenesis factor 2 [Amyelois transitella]	XM_021333273.1	1
PREDICTED: probable 2-oxoglutarate dehydrogenase E1 component DHKTD1 homolog, mitochondrial [Papilio polytes]	XM_021333391.1	1
farnesoic acid O-methyltransferase [Bombyx mori]	XM_021333409.1	2
retinol-binding protein [Anopheles darlingi]	XM_021333587.1	1
PREDICTED: protein virilizer [Amyelois transitella]	XM_021333706.1	2
fatty acid-binding protein 3 [Helicoverpa armigera]	XM_021333801.1	4
ecdysteroid-regulated protein, partial [Helicoverpa armigera]	XM_021333802.1	2

PREDICTED: angiotensin-converting enzyme-like isoform X1 [Bombyx mori]	XM_021333840.1	1
PREDICTED: protein Skeletor, isoforms D/E isoform X1 [Bombyx mori]	XM_021334380.1	1
PREDICTED: telomere length regulation protein TEL2 homolog [Papilio machaon]	XM_021334385.1	1
protease [Helicoverpa armigera]	XM_021334514.1	1
PREDICTED: synaptic vesicle glycoprotein 2B-like [Amyelois transitella]	XM_021334555.1	1
polycalin [Helicoverpa armigera]	XM_021334936.1	3
PREDICTED: nuclear RNA export factor 1 [Bombyx mori]	XM_021334941.1	1
PREDICTED: adenylate cyclase type 8 [Papilio xuthus]	XM_021335116.1	1
PREDICTED: protein 4.1 homolog isoform X2 [Amyelois transitella]	XM_021335209.1	1
PREDICTED: conserved oligomeric Golgi complex subunit 4 [Amyelois transitella]	XM_021335680.1	2
PREDICTED: vacuolar protein sorting-associated protein 45 isoform X1 [Amyelois transitella]	XM_021335688.1	1
PREDICTED: probable malate dehydrogenase, mitochondrial [Papilio machaon]	XM_021335689.1	1
chymotrypsin-like protease C9 [Heliothis virescens]	XM_021335717.1	5
protease [Helicoverpa armigera]	XM_021335730.1	2
PREDICTED: facilitated trehalose transporter Tret1 [Papilio xuthus]	XM_021335746.1	2
PREDICTED: solute carrier family 2, facilitated glucose transporter member 6-like [Bombyx mori]	XM_021335802.1	1
seminal fluid protein HACP038 [Heliconius erato]	XM_021335830.1	1
60S ribosomal protein L15 [Spodoptera frugiperda]	XM_021335870.1	1
modifier of mdg4, partial [Pararge aegeria]	XM_021335943.1	1
vacuolar ATP synthase subunit E [Bombyx mori]	XM_021335983.1	1
serpin-2 [Spodoptera exigua]	XM_021336300.1	1
PREDICTED: fibrillin-1-like [Amyelois transitella]	XM_021336028.1	3
PREDICTED: high affinity copper uptake protein 1-like isoform X1 [Amyelois transitella]	XM_021336232.1	1
takeout-like protein 3 [Helicoverpa armigera]	XM_021336405.1	1
thioredoxin peroxidase [Helicoverpa armigera]	XM_021336615.1	1
PREDICTED: LOW QUALITY PROTEIN: myosin heavy chain, non-muscle-like [Plutella xylostella]	XM_021336899.1	2
PREDICTED: calmodulin isoform X1 [Papilio polytes]	XM_021336917.1	1
PREDICTED: elongation factor 1-alpha 2 [Bombyx mori]	XM_021337025.1	3
aminopeptidase N [Helicoverpa armigera]	XM_021337080.1	5
aminopeptidase N [Helicoverpa armigera]	XM_021337081.1	4
sodium/solute symporter [Anopheles darlingi]	XM_021337272.1	1
PREDICTED: basement membrane-specific heparan sulfate proteoglycan core protein isoform X3 [Papilio machaon]	XM_021337347.1	12
PREDICTED: ATP synthase subunit alpha, mitochondrial [Papilio polytes]	XM_021337525.1	1
actin 5C, isoform B [Drosophila melanogaster]	XM_021337743.1	7
PREDICTED: ornithine aminotransferase, mitochondrial [Bombyx mori]	XM_021337833.1	1
PREDICTED: trypsin, alkaline C-like [Papilio machaon]	XM_021337856.1	1
trypsin [Heliothis virescens]	XM_021337869.1	1
trypsin-like protease [Helicoverpa armigera]	XM_021337877.1	2
trypsin-like protease [Helicoverpa armigera]	XM_021337879.1	2
PREDICTED: tyrosine-protein phosphatase 99A [Amyelois transitella]	XM_021337994.1	1
cytochrome P450 CYP4M10v2 [Helicoverpa armigera]	XM_021338398.1	1
pancreatic lipase 2 [Mamestra configurata]	XM_021338421.1	3
lipase [Helicoverpa armigera]	XM_021338439.1	3
PREDICTED: serine-rich adhesin for platelets-like isoform X1 [Cephus cinctus]	XM_021338461.1	1
trypsin, partial [Helicoverpa armigera]	XM_021338513.1	2
diverged serine protease [Helicoverpa armigera]	XM_021338518.1	4
PREDICTED: transmembrane protease serine 9-like [Papilio polytes]	XM_021338520.1	3
PREDICTED: synaptosomal-associated protein 25 isoform X1 [Bombyx mori]	XM_021338608.1	1
PREDICTED: protein FAM161A [Amyelois transitella]	XM_021338619.1	1
PREDICTED: G-protein coupled receptor Mth2-like [Papilio polytes]	XM_021338627.1	1
PREDICTED: U4/U6.U5 tri-snRNP-associated protein 2 [Papilio polytes]	XM_021338702.1	1
PREDICTED: myosin heavy chain, muscle isoform X5 [Papilio xuthus]	XM_021338811.1	6
PREDICTED: C-5 sterol desaturase erg32-like [Papilio xuthus]	XM_021338969.1	1
PREDICTED: transcription initiation factor TFIID subunit 4 [Bombyx mori]	XM_021339017.1	1
PREDICTED: protein furry [Amyelois transitella]	XM_021339033.1	1
Borealin, partial [Pararge aegeria]	XM_021339216.1	1
PREDICTED: LOW QUALITY PROTEIN: papilin [Bombyx mori]	XM_021339229.1	3
single domain major allergen 2 protein, partial [Helicoverpa armigera]	XM_021339300.1	1
PREDICTED: tigger transposable element-derived protein 1-like [Cyphomyrmex costatus]	XM_021339425.1	1
PREDICTED: carbonyl reductase family member 4-like [Plutella xylostella]	XM_021339635.1	1
Splicing factor 3A subunit 1 [Papilio xuthus]	XM_021339891.1	1
fructose 1,6-bisphosphate aldolase [Danaus plexippus]	XM_021339924.1	1
AAEL002442-PA [Aedes aegypti]	XM_021340037.1	1
RE1-silencing transcription factor [Papilio machaon]	XM_021340084.1	1
arylphorin [Helicoverpa armigera]	XM_021340131.1	9
arylphorin [Helicoverpa armigera]	XM_021340132.1	9
PREDICTED: CDK5RAP1-like protein [Amyelois transitella]	XM_021340164.1	1
PREDICTED: protein cramped [Papilio machaon]	XM_021340266.1	1
PREDICTED: trypsin-7-like [Bombyx mori]	XM_021340310.1	1
PREDICTED: cytochrome b5 [Bombyx mori]	XM_021340359.1	1
PREDICTED: afadin [Bombyx mori]	XM_021340557.1	1
trypsin T2a [Heliothis virescens]	XM_021340589.1	4
trypsin [Heliothis virescens]	XM_021340592.1	1
trypsin-like protease [Helicoverpa armigera]	XM_021340596.1	6
trypsin-like protease [Helicoverpa armigera]	XM_021340599.1	8
serine protease [Helicoverpa armigera]	XM_021340600.1	7
trypsin T4 [Heliothis virescens]	XM_021340602.1	3
Cyclic nucleotide-gated channel rod photoreceptor subunit alpha [Papilio machaon]	XM_021340607.1	2
proline synthetase co-transcribed bacterial-like protein [Bombyx mori]	XM_021340969.1	1
PREDICTED: titin [Bombyx mori]	XM_021341017.1	3
fatty acid-binding protein 2 [Helicoverpa armigera]	XM_021341051.1	5
fatty acid-binding protein 1 [Helicoverpa armigera]	XM_021341061.1	2
fatty acid-binding protein 2 [Helicoverpa armigera]	XM_021341064.1	1
heat shock protein 90 [Helicoverpa armigera]	XM_021341131.1	2
PREDICTED: structure-specific endonuclease subunit SLX4 [Papilio xuthus]	XM_021341178.1	1
chitin deacetylase 5a [Helicoverpa armigera]	XM_021341184.1	4
PREDICTED: aquaporin AQPAe.a [Papilio polytes]	XM_021341250.1	1

PREDICTED: microtubule-actin cross-linking factor 1, isoforms 1/2/3/5 [Amyelois transitella]	XM_021341213.1	1
mitochondrial NADH dehydrogenase iron-sulfur protein 2 [Spodoptera litura]	XM_021341326.1	1
Myrosinase 1 [Papilio xuthus]	XM_021341387.1	1
PREDICTED: golgin subfamily A member 4-like isoform X2 [Papilio xuthus]	XM_021341427.1	1
PREDICTED: nesprin-1 isoform X2 [Amyelois transitella]	XM_021341577.1	2
-	XM_021341648.1	1
apolipophorin precursor protein [Bombyx mori]	XM_021341851.1	19
PREDICTED: histone H2A.V [Plutella xylostella]	XM_021341894.1	2
Repat39-like protein, partial [Heliothis virescens]	XM_021341989.1	4
Repat39-like protein, partial [Heliothis virescens]	XM_021341986.1	1
ATM interactor [Papilio machaon]	XM_021342022.1	1
PREDICTED: round spermatid basic protein 1-like protein [Bombyx mori]	XM_021342295.1	1
PREDICTED: moesin/ezrin/radixin homolog 1 isoform X1 [Amyelois transitella]	XM_021342618.1	7
PREDICTED: myb-like protein V isoform X1 [Plutella xylostella]	XM_021342705.1	1
PREDICTED: dynamin-like 120 kDa protein, mitochondrial isoform X1 [Amyelois transitella]	XM_021343034.1	1
PREDICTED: serine protease inhibitor 3 isoform X1 [Bombyx mori]	XM_021343251.1	1
cathepsin O1-like protease [Chilo suppressalis]	XM_021343409.1	1
PREDICTED: myosin-IB isoform X1 [Papilio xuthus]	XM_021343418.1	2
abnormal wing disc-like protein [Spodoptera litura]	XM_021343497.1	3
copper transporter [Bombyx mori]	XM_021343597.1	1
PREDICTED: phosphoinositide 3-kinase regulatory subunit 4 [Papilio polytes]	XM_021343607.1	1
PREDICTED: dol-P-Man:Man(7)GlcNAc(2)-PP-Dol alpha-1,6-mannosyltransferase [Amyelois transitella]	XM_021343637.1	1
PREDICTED: probable cation-transporting ATPase 13A3 isoform X2 [Amyelois transitella]	XM_021343656.1	1
PREDICTED: cytoplasmic aconitate hydratase-like [Papilio machaon]	XM_021343744.1	1
eIF2 alpha subunit [Spodoptera frugiperda]	XM_021343871.1	2
pancreatic lipase 2 [Mamestra configurata]	XM_021343929.1	4
PREDICTED: protein transport protein Sec61 subunit alpha isoform 2 [Amyelois transitella]	XM_021344022.1	1
transferin [Helicoverpa armigera]	XM_021344034.1	2
diverged serine protease [Helicoverpa armigera]	XM_021344422.1	5
serine protease 52 [Mamestra configurata]	XM_021344467.1	2
neutral lipase [Helicoverpa armigera]	XM_021344621.1	2
PREDICTED: 39S ribosomal protein L51, mitochondrial [Amyelois transitella]	XM_021344681.1	1
inactive lipase, partial [Helicoverpa armigera]	XM_021344707.1	1
trypsin 2 [Helicoverpa armigera]	XM_021344969.1	1
peroxiredoxin-4 precursor [Bombyx mori]	XM_021344991.1	2
PREDICTED: parafibromin [Bombyx mori]	XM_021345005.1	1
PREDICTED: protocadherin-23-like [Plutella xylostella]	XM_021345210.1	3
beta-1,3-glucanase [Helicoverpa armigera]	XM_021345260.1	2
PREDICTED: tropomyosin-2 isoform X3 [Papilio xuthus]	XM_021345380.1	2
PREDICTED: protein kish-A [Amyelois transitella]	XM_021345425.1	1
chymotrypsin [Helicoverpa armigera]	XM_021345778.1	5
chymotrypsin-like protease [Helicoverpa armigera]	XM_021345779.1	7
serine protease 3, partial [Helicoverpa zea]	XM_021345780.1	8
chymotrypsinogen [Helicoverpa punctigera]	XM_021345781.1	7
chymotrypsin-like protease C8 [Heliothis virescens]	XM_021345791.1	1
chymotrypsin-like protease [Helicoverpa armigera]	XM_021345818.1	2
chymotrypsin-like protease [Helicoverpa armigera]	XM_021345819.1	1
PREDICTED: phosphatidylinositol 4-phosphate 5-kinase type-1 gamma isoform X1 [Bombyx mori]	XM_021345965.1	1
Histone H4 [Larimichthys crocea]	XM_021346040.1	4

Table S3. Primers used for qRT-PCR.

Primer name	Sequence (5' to 3')
Trypsin T4-F	TATTGCGGCTCTTCTCTA
Trypsin T4-R	CGGTTGTTGATGATGGTA
Trypsin -F	CATCGTCCGTGAGAACAA
Trypsin -R	ACAACCTCGTTATCATCCAA
esterase E4-F	GCCTTCATACACCTTAGA
esterase E4-R	TTACCAGATTCAGAGACAA
ABCG23-F	ATTGTATTGGTCTAATGAG
ABCG23-R	CATCTTCTAAGGATATGC
ABCC1-F	TTAAGTTGGTTAGTTCAATCAG
ABCC1-R	CCGCATACTCCTTCATAC
Polycalin-F	GAGTCATAAGCACAGATAA
Polycalin-R	GATATAATCCGTTGAGAGTA
APN-F	CTGGATATGTTGAAGAGAA
APN-R	GTAGTAGTCGTAGTCGTA
ALP2-F	ATACTCATCACCACCTCAA
ALP2-R	GAATAGCCACCTCTGTCA
β-Actin-F	CCTGGTATTGCTGACCGTATGC
β-Actin-R	CTGTTGGAAGGTGGAGAGGGAA
